# Actin-binding domains mediate the distinct distribution of two *Dictyostelium* Talins through different affinities to specific subsets of actin filaments during directed cell migration

**DOI:** 10.1371/journal.pone.0214736

**Published:** 2019-04-04

**Authors:** Masatsune Tsujioka, Taro Q. P. Uyeda, Yoshiaki Iwadate, Hitesh Patel, Keitaro Shibata, Tenji Yumoto, Shigenobu Yonemura

**Affiliations:** 1 Electron Microscope Laboratory, RIKEN, Center for Developmental Biology, 2-2-3 Minatojima-minamimachi, Chuo-ku, Kobe, Japan; 2 Department of Physics, Faculty of Science and Technology, Waseda University, Tokyo, Japan; 3 Faculty of Science, Yamaguchi University, Yamaguchi, Japan; 4 Edinburgh Cancer Research Centre, The University of Edinburgh, Crewe Road South, Edinburgh, Scotland; 5 Biomedical Research Institute, National Institute of Advanced Industrial Science and Technology (AIST), Hyogo, Japan; Thomas Jefferson University, UNITED STATES

## Abstract

Although the distinct distribution of certain molecules along the anterior or posterior edge is essential for directed cell migration, the mechanisms to maintain asymmetric protein localization have not yet been fully elucidated. Here, we studied a mechanism for the distinct localizations of two *Dictyostelium* talin homologues, talin A and talin B, both of which play important roles in cell migration and adhesion. Using GFP fusion, we found that talin B, as well as its C-terminal actin-binding region, which consists of an I/LWEQ domain and a villin headpiece domain, was restricted to the leading edge of migrating cells. This is in sharp contrast to talin A and its C-terminal actin-binding domain, which co-localized with myosin II along the cell posterior cortex, as reported previously. Intriguingly, even in myosin II-null cells, talin A and its actin-binding domain displayed a specific distribution, co-localizing with stretched actin filaments. In contrast, talin B was excluded from regions rich in stretched actin filaments, although a certain amount of its actin-binding region alone was present in those areas. When cells were sucked by a micro-pipette, talin B was not detected in the retracting aspirated lobe where acto-myosin, talin A, and the actin-binding regions of talin A and talin B accumulated. Based on these results, we suggest that talin A predominantly interacts with actin filaments stretched by myosin II through its C-terminal actin-binding region, while the actin-binding region of talin B does not make such distinctions. Furthermore, talin B appears to have an additional, unidentified mechanism that excludes it from the region rich in stretched actin filaments. We propose that these actin-binding properties play important roles in the anterior and posterior enrichment of talin B and talin A, respectively, during directed cell migration.

## Introduction

Directed cell migration underlies diverse biological processes including development, wound healing, cancer metastasis, and the immune response [1]. When cells move forward, they extend their front edge by actin polymerization and form nascent adhesion sites on the substrates [1]. Cells acquire traction forces through the adhesion sites which are generally termed focal adhesions in mammals [2]. In contrast, focal adhesions disassemble near the lagging edge, and the contraction of bundles composed of actin and myosin II filaments leads to retraction of the rear [3, 4]. Distinct distributions of molecules involved in these events are maintained during directed cell migration. Several mechanisms mediate the differential localizations of relevant molecules, as shown in the following examples. PI3K generates PtdIns(3,4,5)P_3_ within the plasma membrane along the leading edge, recruiting proteins such as PKB/Akt that harbor the pleckstrin homology (PH) domain, which binds to PtdIns(3,4,5)P_3_ [5]. A number of actin-binding proteins are recruited to the leading edge by their interaction with actin filaments [1]. Phosphorylation of *Dictyostelium* myosin II by its specific kinase, MHCK, which is localized along the leading edge, causes disassembly of myosin II filaments in the anterior cortical region, enhancing the posterior localization of myosin II [6]. The cytoskeletal cortex, which is mainly composed of actin filaments and associated proteins such as myosin II, flows backward during directed cell migration, and transports cell surface markers linked to the cortex toward the rear end [7, 8]. *Dictyostelium* PTEN associates with the plasma membrane more stably along the lagging edge than the leading edge, accounting for its posterior enrichment [9]. It is well known that PAR proteins regulate cell polarity in various contexts. As examples of directed cell migration, TIAM1, which enhances pseudopod extension via Rac1 activation, is recruited along the leading edge by binding to Par3, a member of the Par protein family [10]. Par6, another PAR protein member, binds aPKC along the leading edge, and the complex recruits Smurf1, an E3 ubiquitin ligase, to the leading edge to degrade RhoA, which mediates acto-myosin contraction [11].

Talin is one of the most important proteins involved in cell migration. It is essential for the activation of integrins, which are adhesion molecules of focal adhesions, and mediates the linkage between integrins and the actin cytoskeleton, thereby transmitting motile forces to the substrate [12, 13]. Talin also links the cell cortex to the plasma membrane [4, 14, 15]. The FERM domain located at the N-terminus of talin binds to membrane lipids and several protein components of focal adhesions, including integrins. Meanwhile, an actin-binding domain termed the I/LWEQ domain is located near the C-terminus. Talin is generally believed to function as a dimer, and the I/LWEQ domain also contains a motif to mediate dimerization [13, 16]. Since a second talin gene was identified in vertebrates [17], the unique roles of each of the two talins, talin1 and talin2, have been addressed [18–20]. Distinct sub-cellular localizations were recently reported in several cell lines such as mouse fibroblasts and rat aortic smooth muscle cells, in which talin1 and talin2 are enriched in peripheral and central focal adhesions, respectively [21]. Biochemically, both talins bind integrins and actin filaments but with different affinities [22, 23].

*Dictyostelium* is the first organism in which a second talin gene was discovered, and the two talin genes encode talin A and talin B, respectively [24, 25]. Distinct from other talins including talin A, talin B has a unique C-terminal extension, in which a potential actin-binding domain homologous to villin headpiece (VHP) is linked to the C-terminus of the I/LWEQ domain via a short proline-rich region (PRR) [25, 26, 27]. Upon starvation, solitary *Dictyostelium* amoebae aggregate into hemispherical multi-cellular structures, which then transform to fruiting bodies. In the process of aggregation, individual amoebae show robust directed locomotion towards aggregation centers by chemotaxis to cAMP, forming cell streams comprised of highly polarized cells. Talin A is enriched in the posterior region of chemotaxing cells and assists the detachment of adhesion molecules from the substrate and the rear retraction [4]. The association of talin A with actin filaments through its I/LWEQ domain may determine the rear distribution [4]. In this study, we examined the distribution of talin B in streaming cells and found that it was enriched along the leading edge. The distinct distributions of the two *Dictyostelium* talins prompted us to elucidate the underlying mechanism since it would also provide insight into the general mechanism of distinct molecular distributions in polarized cells. Our results suggest that different affinities of the actin-binding regions of the two talins to specific subsets of actin structures play important roles in their distinct distributions.

## Materials and methods

### Generation of expression constructs

To generate the construct to express GFP-talin B, the partial fragment of *talB* cDNA between the unique *Sac*I site and the termination codon was obtained from the construct of FLAG-tagged talin B [28] using *Sac*I and *Xba*I, and was subcloned into the pTX-GFP vector [29]. Subsequently, the fragment from the fourth nucleotide to the *Sac*I site of the *talB* gene amplified by genomic PCR was inserted into the plasmid to complete the GFP-talin B construct. The construct to express talin A-RFP was generated by replacing the gene coding for GFP with that coding for mRFP in the talin A-GFP construct [4].

To generate the constructs to express GFP-I/LWEQ(talA), GFP-I/LWEQ(talB), GFP-I/LWEQ(talB)-PRR-VHP, and GFP-PRR-VHP, the cDNA fragments coding for amino acid residues 2255 to 2492 of talin A, 2225 to 2457 of talin B, 2225 to 2614 of talin B, and 2458 to 2614 of talin B were amplified by PCR using the talin A-GFP construct [28] or the GFP-talin B construct as a template, and were subcloned into pTX-GFP as *Sac*I/*Xba*I fragments. To generate the construct for GFP-I/LWEQ(talA)-PRR-VHP, the cDNA fragment coding for amino acid residues 2458 to 2614 of talin B was inserted into the *Xba*I site of the construct of GFP-talA-I/LWEQ as an *Xba*I fragment.

To generate the construct to express GST-FERM in *E*. *coli*, the cDNA fragment coding for amino acid residues 81 to 280 of talin B was amplified and subcloned into pGEX-6-P3 vector (GE Healthcare) as a *Sal*I/*Not*I fragment. To generate the constructs to express GST-I/LWEQ(talA), GST-I/LWEQ(talB), and GST-I/LWEQ(talB)-PRR-VHP in *E*. *coli*, the cDNA fragments coding for amino acid residues 2255 to 2492 of talin A, 2225 to 2457 of talin B, and 2225 to 2614 of talin B were amplified by PCR using the talin A-GFP construct [28] or the GFP-talin B construct as a template, and were subcloned into pGEX-6-P3 vector as a *Bam*HI/*Eco*RI fragment for I/LWEQ(talA), a *Bam*HI/*Xho*I fragment for I/LWEQ(talB), and a *Bam*HI/*Xho*I fragment for I/LWEQ(talB)-PRR-VHP.

### Strains and conditions for growth and development

Strains used in the present study were *Dictyostelium* Ax2 as the wild-type strain, HG1664 as the talin A-null strain [24], HKT104 as the talin B-null strain [12], a strain lacking both talin A and talin B (talin A-/talin B-null) [28], HS1 as the myosin II-null strain [30], HS1 lacking talin A (myosin II-/talin A-null), and HS1 lacking talin B (myosin II-/talin B-null). All strains were grown axenically in axenic HL5 medium [31] at 22°C in plastic Petri dishes. The plasmids to express the GFP-PH domain of CRAC (WF38) (kindly provided by Dr. Y. Kamimura, Johns Hopkins University) [32], GFP-myosin II [33], mCherry-actin [33], and GFP or RFP fusion proteins of entire or part of the talins used in this study were introduced into wild-type, talin A-null, talin B-null, talin A/talin B-null, myosin II-/talin A-null, or myosin II-/talin B-null strains separately or simultaneously by electroporation. The expression of all fluorescent proteins was driven by the constitutive actin 15 promoter. The transformants expressing GFP-talin B, talin A-GFP, or both GFP-talin B and talin A-RFP were selected in axenic HL5 medium containing 30 μg/ml G418, and the other transformants were selected by 10 μg/ml G418. Cells were allowed to develop at 22°C on 1% non-nutrient agar buffered with KK_2_ phosphate (16.5 mM KH_2_PO_4_, 3.8 mM K_2_HPO_4_, pH 6.2).

### Confocal microscopy

In the following three experiments, fluorescence images were acquired by a Leica DMIRE2 microscope with a confocal unit (CSU10; Yokogawa) and a CCD camera (SensiCam QE; Cooke) controlled by MetaMorph Imaging software, version 7.0r4 (Molecular Devices), and a HCX PL APO 63x/1.40 NA oil Ph3 lens.

To observe the distributions of GFP fusion proteins in cells with disrupted actin structures in a cAMP gradient, we prepared starved cells expressing GFP-talin B or talin A-GFP, and the experiment was set up as previously performed for a cell motility assay using a micro-capillary [4], except that latrunculin A (Calbiochem), an agent that disrupts actin structures, was added to the buffer at a final concentration of 3 μM. Briefly, the starved cells suspended in KK2 phosphate buffer containing 3 μM latrunculin A were transferred to glass-bottom dishes (35 mm; Iwaki). The tip of a micro-capillary tube (Sterile Femtotips; Eppendorf) filled with 1 mM cAMP was placed near the glass surface, and fluorescent images of the cells were acquired by confocal microscopy.

Time lapse imaging of aggregating cells expressing GFP fusion proteins in the streams was performed as described previously [4]. For time lapse imaging of the vegetative cells, cells growing in HL5 were transferred to glass-bottom dishes and imaged. In both cases, fluorescent images were captured at 5 s intervals using confocal microscopy.

For time lapse imaging of cells globally stimulated with cAMP, the starved cells expressing GFP fusion proteins were suspended in 250 μl KK2 phosphate buffer and the cell suspensions were transferred to glass-bottom dishes. The same volume of KK2 phosphate buffer containing 2 μM cAMP was added to the cells for global stimulation. Fluorescent images were taken every second starting from two seconds before the stimulation using confocal microscopy.

### Membrane-lipid binding assay

A biochemical assay to explore the lipid-binding ability of the FERM domain of talin B was performed as described previously [4]. Briefly, the GST-tagged FERM domain was expressed in *E*. *coli* strain BL21 (pLys). Membrane Lipid Strips (P-6002; Echelon Biosciences) were incubated in a bacterial lysate for 12 h at 4°C. The GST-FERM domain bound to lipids spotted on the strips was detected by a chemiluminescence assay (Immobilon Western; Millipore) using an anti-GST monoclonal antibody (B-14; Santa Cruz) and a horseradish peroxidase (HRP)-conjugated anti-mouse secondary antibody (Jackson ImmunoResearch).

### Aspiration assay

Suction was applied to migrating cells as described previously [33]. Briefly, a suction pipette with an inner diameter of 3 μm was made from a glass capillary (G-1, Narishige, Tokyo, Japan) using a pipette puller (PG-1, Narishige) and a microforge (MF-830, Narishige). The pipette was then connected to a vertical open-ended glass tube and a 5 ml syringe via a silicone tube. The syringe was then used to adjust the height of the water surface in the glass tube so that the hydrostatic pressure at the mouth of the suction pipette was 2.5 kPa.

### Observation of fixed cells

Observation of fixed cells undergoing cytofission was essentially accomplished by a procedure described previously [33]. Briefly, myosin II-/talin A-null cells expressing GFP-I/LWEQ(talA), GFP-I/LWEQ(talA)-PRR-VHP, or talin A-GFP and myosin II-/talin B-null cells expressing GFP-I/LWEQ(talB)-PRR-VHP or GFP-talin B were fixed and stained with rhodamine-phalloidin to visualize actin filaments. To quantify relative amounts of the GFP-tagged proteins to actin filaments at specific cellular locations, signal intensities of GFP were divided by those of rhodamine-phalloidin.

### Cosedimentation assay

GST-tagged I/LWEQ(talA), I/LWEQ(talB), and I/LWEQ(talB)-PRR-VHP were separately expressed in *E*. *coli* strain Rosetta (DE3). Cells expressing these proteins were harvested, resuspended in lysis buffer (1× PBS, pH 7.4, 1% Triton X-100, 1 mg/mL lysozyme [Nacalai], 0.5 units/mL DNase I [Takara], and a protease inhibitor cocktail [GE Healthcare]), sonicated, and centrifuged at 10,000 x *g* for 40 min. After the supernatants were incubated with Glutathione-Sepharose 4B (GE Healthcare) for 3 h at 4°C, the resin collected was loaded on a Poly-Prep Chromatography Column (Bio-Rad), and the column was washed with three volumes of washing buffer (1× PBS, pH 7.4, and 1% Triton X-100). Subsequently, the bottom of the column was sealed and the resin was incubated with protease buffer (50 mM Tris-Cl, pH 7.5, 150 mM NaCl, 1 mM EDTA, 1 mM DTT) containing 10 units of PreScission Protease (GE Healthcare) for 22 h at 4°C to cleave off the GST tag from the target proteins. The target proteins were then eluted by unsealing the bottom of the column. Budding yeast fimbrin fused with GST at the N-terminus was expressed in *E*. *coli* and purified using Glutathione-Sepharose 4B. It was then separated from GST by treatment with thrombin followed by ion-exchange chromatography.

I/LWEQ(talA), I/LWEQ(talB), I/LWEQ(talB)-PRR-VHP, and yeast fimbrin were incubated at 1 μM with 5 μM rabbit skeletal muscle F-actin in a buffer containing 10 mM HEPES pH 7.4, 50 mM KCl, 2 mM MgCl_2_, 1 mM ATP and 1 mM DTT for 1 h at 22°C. The mixtures were first centrifuged at 10,000 x *g* for 15 min at 22°C. The supernatants were then ultracentrifuged at 100,000 x *g* for 20 min at 22°C. The pellets after low centrifugation, and the supernatants and pellets after ultracentrifugation were separately loaded to 12% acrylamide gels for SDS-PAGE.

### Immuno-blot analysis

Wild-type cells, talin B-null cells, and transformants expressing each GFP-protein were individually lysed in SDS buffer (50 mM Tris-Cl (pH 6.8), 2% SDS) containing protease inhibitor cocktail (Nacalai) and were heated at 95°C for 5 min. The lysates were then subjected to SDS-PAGE and were transferred to PVDF membranes, which were then incubated with a mouse anti-GFP antibody (Santa Cruz; sc-9996), a mouse anti-actin antibody (Millipore; MAB1501), or a rabbit anti-talin B antiserum [12]. The immune complexes were detected using HRP-conjugated anti-mouse or anti-rabbit secondary antibodies and Chemi-Lumi One Super reagent (Nacalai).

## Results

### Localization of talin B along the leading edge during directed cell migration

**Fig 1:**
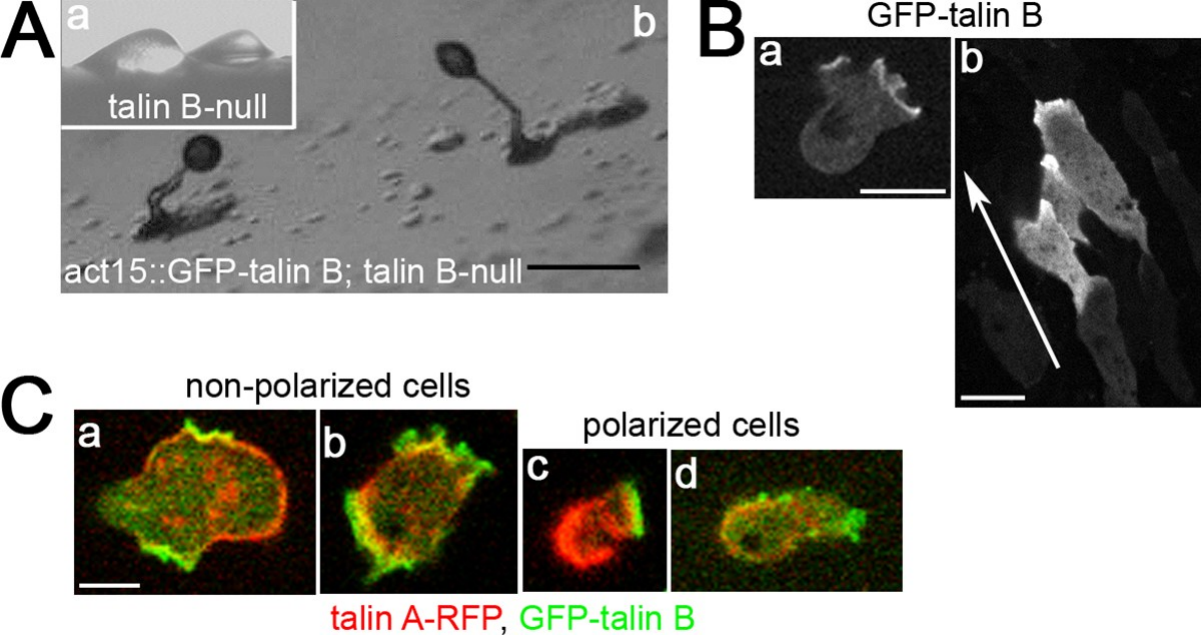
**Sub-cellular localizations of talin A and talin B.** (A) (a) Arrested mounds formed by talin B-null cells. (b) Fruiting bodies formed by talin B-null cells expressing GFP-talin B. The cells were allowed to develop on non-nutrient agar. Although immuno-blot analysis confirmed that the average expression level of GFP-talin B was higher than endogenous talin B in wild-type cells, 40% of the transformed cells did not exhibit the fluorescence signal (S1A and S1B Fig). (B) Confocal images of vegetative (a) and streaming (b) talin B-null cells expressing GFP-talin B. The arrow indicates the direction of migration. In vegetative cells, GFP-talin B accumulated along pseudopodia, often showing a cup-shaped pattern, which is characteristic of macropinocytotic crowns [35]. In streaming cells, GFP-talin B was concentrated along the leading edge. Note that the cells clearly showing localization of GFP-talin B were surrounded by other cells with little or no fluorescence signals in the aggregation stream. (C) Confocal images of vegetative talin A-/talin B-null cells simultaneously expressing talin A-RFP and GFP-talin B. Cells shown in (a) and (b) were apparently non-polarized, whereas those shown in (c) and (d) were polarized. Scale bars: 200 μm (A) and 5 μm (B,C).

In comparison with the posterior enrichment of talin A during directed cell migration reported previously [4], the sub-cellular distribution of talin B, another *Dictyostelium* talin homologue, was explored. We generated a construct to express talin B tagged N-terminally with GFP (GFP-talin B) under the control of the actin15 promoter, and introduced it into talin B-null cells. Immuno-blot analysis confirmed the expression of GFP-talin B in the transformed cells, whose level was higher than that of endogenous talin B in wild-type cells (S1A Fig). As reported previously [25], the development of talin B-null cells was arrested at the mound stage, but the expression of GFP-talin B partially reverted this phenotype and allowed the talin B-null cells to form small fruiting bodies (Fig 1Aa,b). Although the average expression level of GFP-talin B was sufficient as assayed by immuno-blot analysis, fluorescence microscopy revealed that the expression levels of individual transformed cells were highly variable and approximately 40% of the cells hardly exhibited fluorescence signals even after drug selection (S1B Fig), which is likely to cause incomplete restoration of the development of talin B-null cells. In accordance with this observation, a similar developmental phenotype was observed in the mixed development of wild-type and talin B-null cells [12].

A cup-shaped staining pattern and the accumulation of GFP-talin B along pseudopodia in vegetative cells were similar to those reported previously (Fig 1Ba) [34]. The distributions around the regions rich in actin filaments are also consistent with anti-talin B antibody staining of vegetative cells previously reported [12]. We observed the localization of GFP-talin B in streaming cells and found that it was localized along their leading edge (Fig 1Bb and S1 Movie), which is in contrast to talin A. This distribution is similar to its anterior accumulation in cells at a later developmental stage, as was revealed previously by anti-talin B antibody staining [12].

We previously showed that talin A was also enriched in pseudopodia in vegetative cells, which were non- or poorly polarized compared with chemotaxing cells [4]. We therefore carefully compared the localizations of talin A and talin B within the same vegetative cells. This was achieved by simultaneously introducing constructs to express GFP-talin B and talin A tagged with mRFP (talin A-RFP), which has been reported to restore the defects of talin A-null cells and show a similar staining pattern to the antibody staining [4], into cells lacking both talin A and talin B (talin A-/talin B-null cells). We found that talin A-RFP was distributed throughout a greater part of the cell peripheries, while the GFP-talin B signal was restricted to rough peripheries, including outward extensions presumably driven by actin polymerization (Fig 1Ca,b). Consistent with their localization in streaming cells, spontaneously polarized vegetative cells exhibited clear anterior and posterior enrichment of GFP-talin B and talin A-RFP, respectively (Fig 1Cc,d) [4].

### Lipid-binding specificity of the FERM domain of talin B

**Fig 2:**
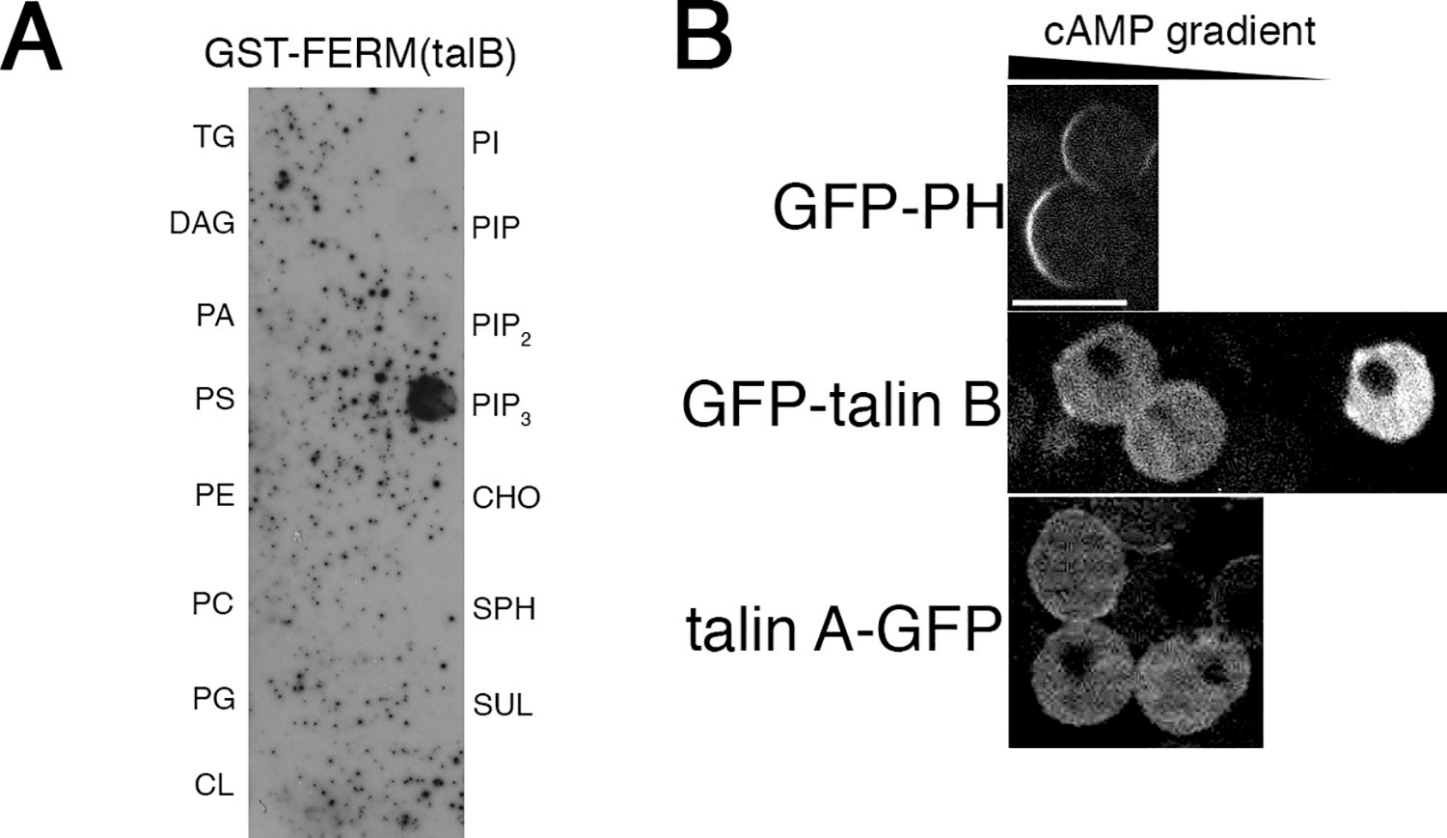
Biochemical properties of the talin B FERM domain and requirement of actin filaments for the talin B localization. (A) Biochemical assay to determine the binding specificities of the FERM domain of talin B to the plasma membrane components. A bacterial lysate containing GST-FERM was reacted with a membrane strip spotted with 15 unique lipids. GST-FERM bound to the indicated lipids was immunologically detected. TG, triglyceride; DAG, diacylglycerol; PA, phosphatidic acid; PS, phosphatidylserine; PE, phosphatidylethanolamine; PC, phosphatidylcholine; PG, phosphatidylglycerol; CL, cardiolipin; PI, phosphatidylinositol; PIP, PtdIns(4)P; PIP_2_, PtdIns(4,5)P_2_; PIP_3_, PtdIns(3,4,5)P_3_; CHO, cholesterol; SPH, sphingomyelin; and SUL, 3-sulfogalactosylceramide. (B) Sub-cellular localizations of the GFP-PH domain of CRAC, a probe for PtdIns(3,4,5)P_3_, as well as GFP-talin B and talin A-GFP, in the cAMP gradient after treatment of starved cells with latrunculin A. Scale bar: 10 μm.

To gain insight into the molecular mechanism of the distinct sub-cellular distributions of talin A and talin B, we first compared the properties of their FERM domains. The FERM domain is a conserved domain at the N-terminus of talin, and is generally known to bind PtdIns(4,5)P_2_ (phosphatidylinositol 4,5-bisphosphate) [13]. We previously reported that the FERM domain of talin A binds PtdIns(4,5)P_2_ and PtdIns(3,4,5)P_3_ (phosphatidylinositol 3,4,5-triphosphate) in a biochemical assay, while talin A itself was suggested to bind PtdIns(4,5)P_2_ *in vivo* [4]. Thus, the same biochemical assay was performed against the FERM domain of talin B. A membrane strip on which 15 types of plasma membrane lipids were spotted was incubated with a lysate of bacteria expressing the GST-tagged FERM domain of talin B (GST-FERM). We found that GST-FERM of talin B bound exclusively to PtdIns(3,4,5)P_3_ (Fig 2A), unlike the FERM domain of talin A that binds both PtdIns(4,5)P_2_ and PtdIns(3,4,5)P_3_ [4], indicating different lipid binding specificities between the FERM domains of talin A and talin B.

### Requirement of actin filaments for the specific sub-cellular localization of talin B

We next examined if the sub-cellular localization of GFP-talin B was determined by the specific binding of the talin B FERM domain to PtdIns(3,4,5)P_3_. Even when the starved cells lost their morphological polarity by treatment with latrunculin A, an agent that disrupts actin structures, the asymmetric sub-cellular distribution of PtdIns(3,4,5)P_3_ was still maintained in the cAMP gradient, such that PtdIns(3,4,5)P_3_ was concentrated within the plasma membrane facing the cAMP source (Fig 2B, top) [32, 36]. Under that condition, the distribution of GFP-talin B was diffuse throughout the cytoplasm (Fig 2B, middle), indicating that actin filaments are required for the asymmetric localization of talin B and that binding of the talin B FERM domain to PtdIns(3,4,5)P_3_ is insufficient for the sub-cellular localization of talin B. Talin A-GFP was also diffusely distributed across the cytoplasm under this condition (Fig 2B, bottom), confirming the requirement of actin filaments for the specific sub-cellular localization of talin A, as we reported previously [4].

### Biochemical actin-binding properties of the C-terminal domains of talin A and talin B

**Fig 3:**
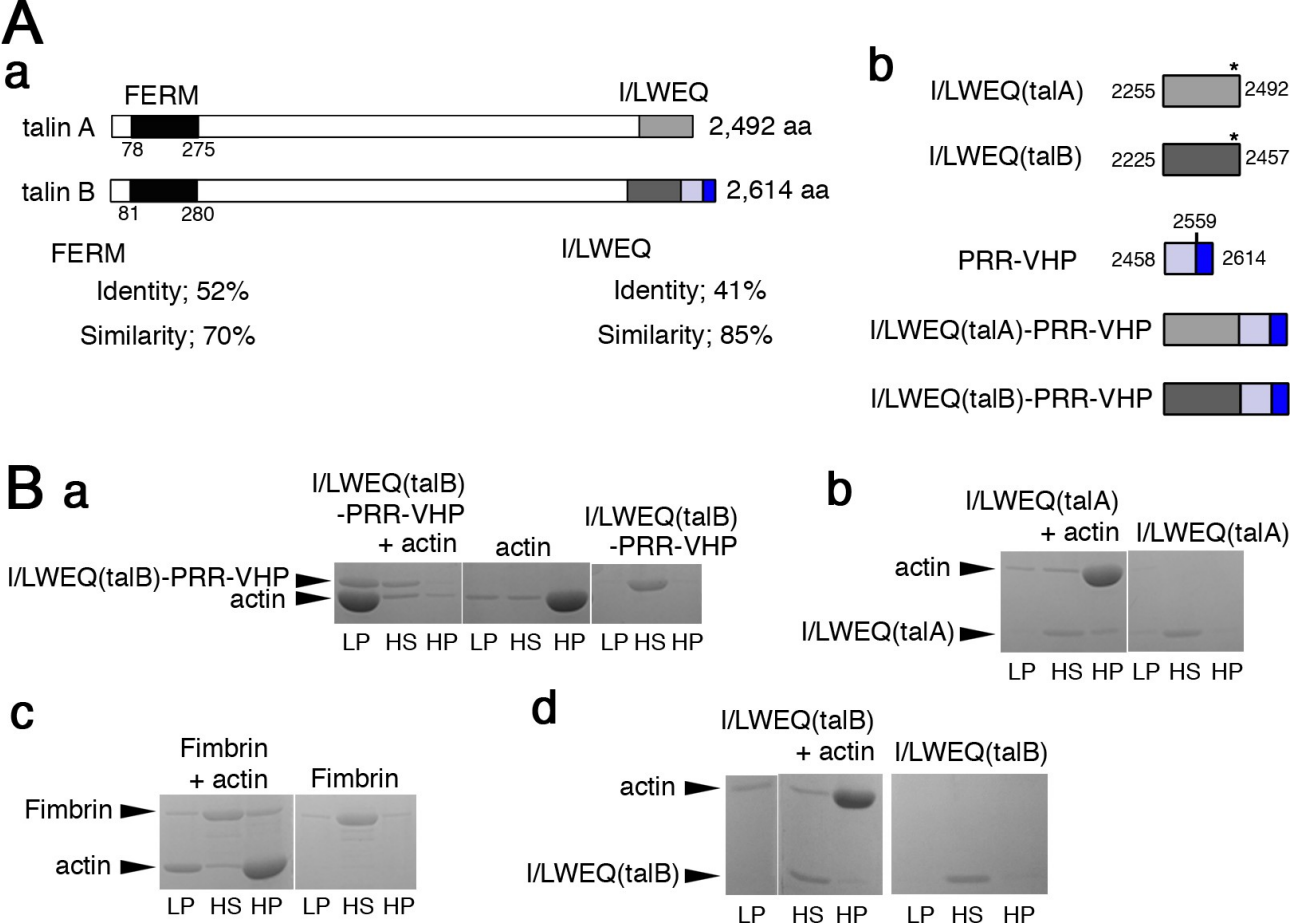
Biochemical analysis of the actin-binding domains of talin A and talin B. (A) (a) Schematic representations of talin A and talin B. The FERM domains of both talins are shown in black, I/LWEQ(talA) and I/LWEQ(talB) in light and dark gray, respectively, the proline-rich region in light blue, the VHP domain in blue. Sequence identities and similarities between the two FERM domains as well as the two I/LWEQ domains were calculated with the Blast2 program and are indicated under the illustrations. (b) Schematic representations show boundaries of C-terminal domains in each talin and structures of C-terminal talin fragments. Each domain is indicated by different colors as shown in (a). Numbers refer to amino acid positions of the boundaries. Asterisks indicate approximate positions of the dimerization motifs in I/LWEQ. The sequence alignment between I/LWEQ(talA) and I/LWEQ(talB) is displayed in S2A Fig, which also shows key amino acid residues for dimerization [16]. (B) Cosedimentation of I/LWEQ(talB)-PRR-VHP (a), I/LWEQ(talA) (b), Fimbrin (c), and I/LWEQ(talB) (d) with actin filaments was examined by low and high speed centrifugation. Sedimentation of each actin-binding protein without actin was also tested. Pellet samples after low and high speed centrifugations (LP and HP) and supernatant samples after high speed centrifugation (HS) were analyzed by SDS-PAGE.

The I/LWEQ domain is the C-terminal actin-binding domain that is conserved in talin (Fig 3Aa and S2A Fig). It was reported that GFP fusion of the I/LWEQ domain of talin A (GFP-I/LWEQ(talA)) exhibits the posterior enrichment as talin A itself, leading to the conclusion that the actin-binding domain determines the sub-cellular distribution of the whole protein [4]. We thus analyzed the C-terminal actin-binding region of talin B. We initially verified the actin-binding activity of the C-terminal region, which is comprised of the I/LWEQ domain connected to the VHP domain via a short proline-rich sequence (I/LWEQ(talB)-PRR-VHP) (Fig 3Aa,b), by cosedimentation assays. GST-tagged I/LWEQ(talB)-PRR-VHP was expressed in bacteria and then GST was cleaved off from I/LWEQ(talB)-PRR-VHP in the process of its purification. More than half of total I/LWEQ(talB)-PRR-VHP cosedimented with filaments of rabbit skeletal muscle actin by low speed centrifugation (Fig 3Ba). Pelleting of actin filaments alone required high speed centrifugation, while I/LWEQ(talB)-PRR-VHP alone was not pelleted even by high speed centrifugation (Fig 3Ba). This suggests that I/LWEQ(talB)-PRR-VHP not only binds actin filaments directly but also bundles the filaments [37]. I/LWEQ(talA) (Fig 3Aa,b), in contrast, cosedimented with actin filaments only by high speed centrifugation (Fig 3Bb), confirming the previous finding that it lacks actin-bundling activity [38]. Yeast fimbrin, which was used as a control protein harboring actin-bundling activity [39], partially cosedimented with actin filaments by low speed centrifugation (Fig 3Bc). Notably, the bundling activity of I/LWEQ(talB)-PRR-VHP seemed to be stronger than that of fimbrin because the fraction of actin filaments pelleted with I/LWEQ(talB)-PRR-VHP by low speed centrifugation was much larger than that with fimbrin (Fig 3Ba,c). We also explored the actin-binding activity of I/LWEQ(talB) alone (Fig 3Aa,b). In contrast to I/LWEQ(talB)-PRR-VHP, I/LWEQ(talB) did not cosediment with actin filaments during low speed centrifugation (Fig 3Bd). Even after high speed centrifugation, its cosedimentation with actin filaments was hardly detectable, implying that the VHP domain is essential for the high actin-binding affinity and the actin-bundling properties of I/LWEQ(talB)-PRR-VHP.

### Sub-cellular localizations of the C-terminal actin-binding domains of talin A and talin B

**Fig 4:**
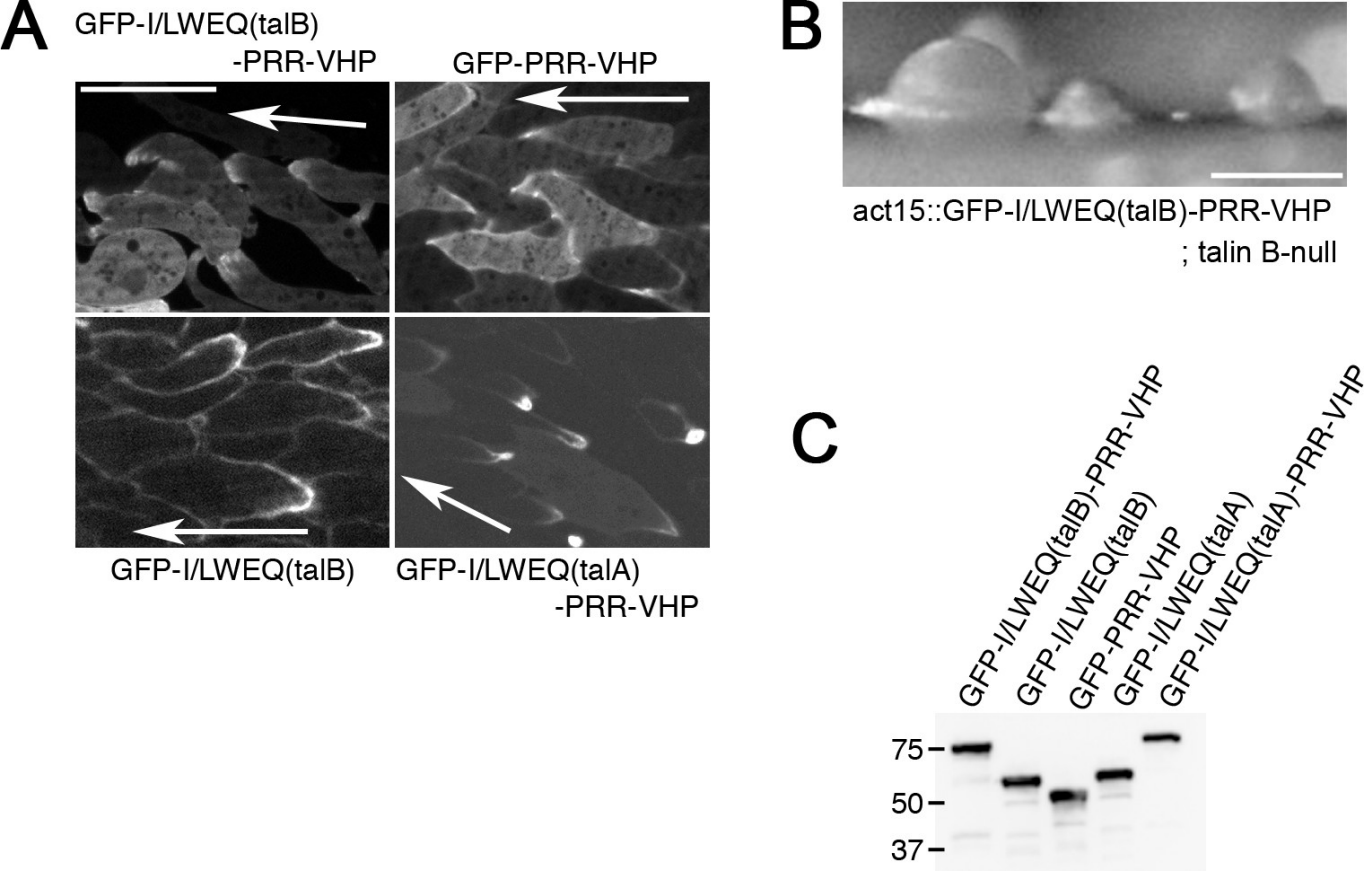
**Sub-cellular localizations of C-terminal actin-binding domains of talin A and talin B.** (A) Confocal images of streaming talin B-null cells expressing GFP-I/LWEQ(talB)-PRR-VHP, GFP-I/LWEQ(talB), or GFP-PRR-VHP and talin A-null cells expressing GFP-I/LWEQ(talA)-PRR-VHP. Arrows indicate the direction of migration. GFP-PRR-VHP and GFP-I/LWEQ(talA)-PRR-VHP were also observed in wild-type cells (shown in S2B Fig). (B) Arrested mounds formed by talin B-null cells expressing GFP-I/LWEQ(talB)-PRR-VHP. (C) Immuno-blot analysis using whole cell lysates and an anti-GFP antibody. Marker sizes (kDa) are indicated on the left side of the blot. Scale bars: 10 μm (A) and 200 μm (B).

We subsequently explored the sub-cellular localization of I/LWEQ(talB)-PRR-VHP by GFP fusion. Because different tags or linkers originating from different expression vectors reportedly affect the sub-cellular localization of certain actin-binding domains [40], we used the same expression vector as that used for the expression of GFP-I/LWEQ(talA) (Fig 3Aa,b) [4], which harbors a polyhistidine tag at the N-terminus of GFP. Since the I/LWEQ domain of talin possesses an element to mediate its dimerization (Fig 3Ab and S2A Fig) [16], GFP-I/LWEQ(talB)-PRR-VHP may form a hetero-dimer with endogenous full length talin B in wild-type cells. This could affect the localization of GFP-I/LWEQ(talB)-PRR-VHP. Therefore, we used talin B-null cells to express this region. This would also avoid the GFP fusion protein from competing with talin B for the binding to actin filaments. In streaming cells, GFP-I/LWEQ(talB)-PRR-VHP localized along the leading edge (Fig 4A and S2 Movie), which was indistinguishable from GFP-talin B. Actin structures were required for asymmetric distribution of talin B and the co-sedimentation assays demonstrated direct binding between actin filaments and I/LWEQ(talB)-PRR-VHP (Fig 3Ba), so the anterior localization of talin B during directed cell migration appears to be mediated by the association of I/LWEQ(talB)-PRR-VHP with actin filaments. Albeit the same sub-cellular localization as that of GFP-talin B, GFP-I/LWEQ(talB)-PRR-VHP was not able to rescue the developmental defect of talin B-null cells (Fig 4B).

To observe the localization of the two potential actin-binding domains of talin B separately, we fused GFP to the I/LWEQ module (GFP-I/LWEQ(talB)) and to the VHP domain with the preceding proline-rich region (GFP-PRR-VHP) separately (Fig 3Ab). GFP-I/LWEQ(talB) accumulated along the lagging edge while GFP-PRR-VHP concentrated along the leading edge in talin B-null cells (Fig 4A, S3 and S4 Movies) as well as in wild-type cells (S2B Fig). These results imply that the VHP domain is a key element for targeting talin B to the leading edge. We also expressed GFP fused to I/LWEQ(talA)-PRR-VHP chimeric fragment in wild-type and talin A-null cells (Fig 3Ab). In contrast to GFP-I/LWEQ(talB)-PRR-VHP, GFP-I/LWEQ(talA)-PRR-VHP accumulated along the lagging edge (Fig 4A, S2B Fig, and S5 Movie). This observation indicates that, unlike I/LWEQ(talB), I/LWEQ(talA) is capable of overriding the ability of the VHP domain to target to the leading edge.

To confirm that the plasmids used in the above experiments drive expression of expected GFP fusion proteins, we performed immuno-blot analysis using lysates of cells expressing each GFP fusion protein and an anti-GFP antibody. All the proteins were found to migrate at their predicted sizes (Fig 4C), validating the expression of expected proteins.

### Coordinated behaviors of talin A and talin B with myosin II and actin filaments

**Fig 5:**
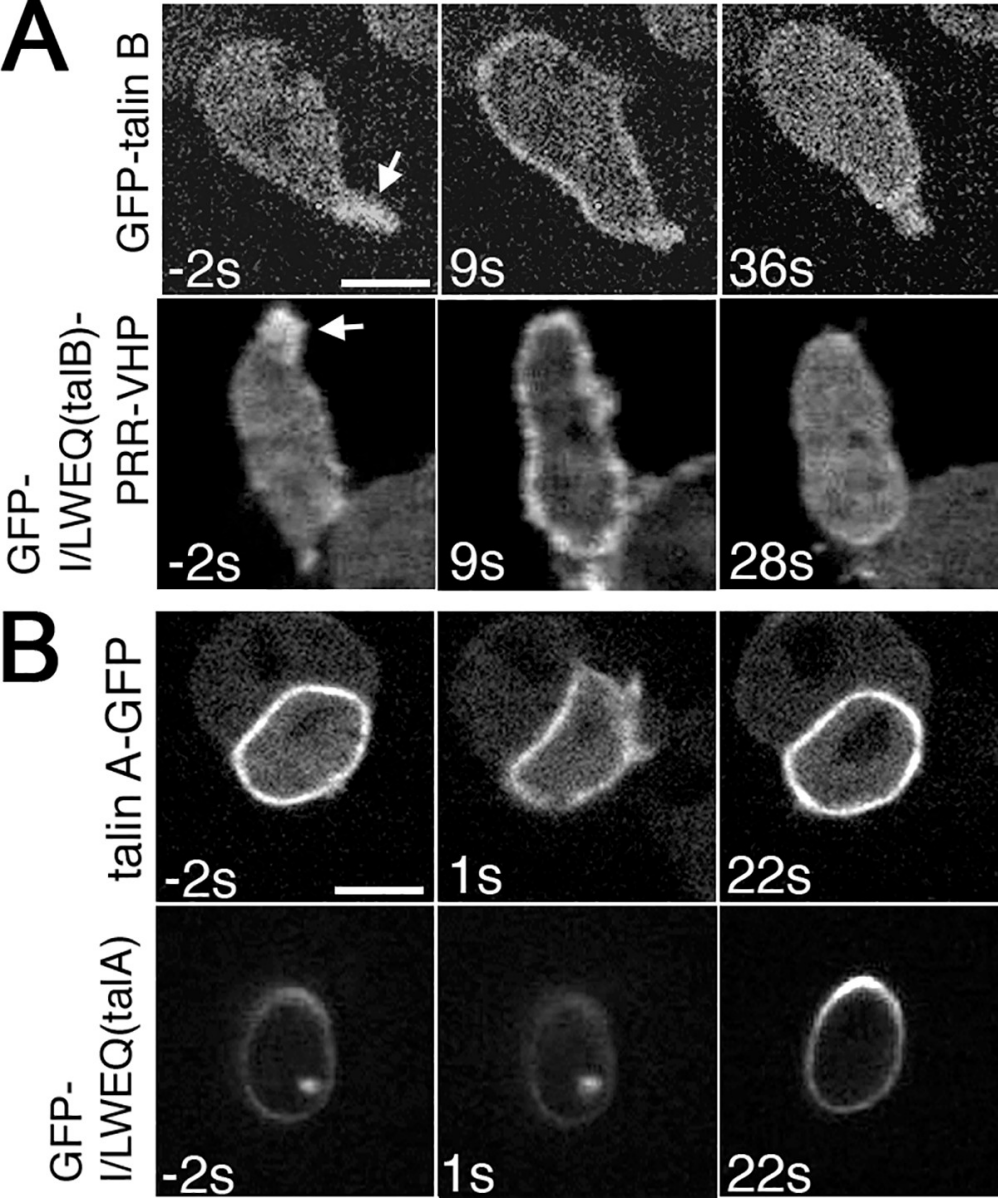
**Responses of talin A, talin B, and their actin-binding domains to global stimulation with cAMP.** Time lapse confocal images of starved talin B-null cells expressing GFP-talin B or GFP-I/LWEQ(talB)-PRR-VHP (A) and talin A-null cells expressing talin A-GFP or GFP-I/LWEQ(talA) (B) taken before and after global stimulation with cAMP. Times before and after stimulation are indicated at the lower left corners in seconds. Arrows indicate the accumulation of GFP-talin B or GFP-I/LWEQ(talB)-PRR-VHP in cell projections. Scale bars: 5 μm.

Active actin polymerization occurs in pseudopodia and in the leading edge of chemotaxing cells [1], where talin B and its C-terminal actin-binding region (I/LWEQ(talB)-PRR-VHP) were enriched. To further investigate the coordination of actin polymerization with talin B and I/LWEQ(talB)-PRR-VHP, we explored the behaviors of talin B and I/LWEQ(talB)-PRR-VHP in starved cells upon uniform exposure to cAMP, which induces temporary actin polymerization throughout the cell peripheries [5]. Prior to stimulation, both GFP-talin B and GFP-I/LWEQ(talB)-PRR-VHP, individually expressed in talin B-null cells, accumulated in cell projections without any prominent distribution along the cortical regions (Fig 5A; arrows). However, their amount along the cortical regions transiently increased and returned to the basal level following cAMP treatment (Fig 5A). This response is reminiscent of transient actin filament formation upon cAMP treatment [5]. We speculate that the association of I/LWEQ(talB)-PRR-VHP with the newly polymerized actin filaments leads to the transient enrichment of talin B along the cell cortex. Reciprocal to actin filament formation upon global cAMP stimulation, myosin II transiently and partially dissociated from the cortical region, followed by reassociation at the same location within 30 s [5]. In light of the above results as well as our previous report suggesting that talin A is associated with acto-myosin structures through its I/LWEQ domain (I/LWEQ(talA)) [4], we also explored whether talin A and I/LWEQ(talA) would behave similarly to myosin II upon cAMP stimulation. Both talin A-GFP and GFP-I/LWEQ(talA) individually expressed in talin A-null cells distributed along the cortical region before stimulation (Fig 5B). After the addition of cAMP, both GFP fusion proteins transiently dissociated from the cortex and returned there subsequently (Fig 5B), similar to myosin II. Notably, their signal intensities along the cortical region after reassociation were higher than those before stimulation (Fig 5B). This is also similar to the response of myosin II after cAMP stimulation [5]. From these results, we propose a model in which enrichment of actin filaments by active polymerization determines the sub-cellular localization of talin B via the association with its C-terminal actin-binding domain, while talin A associates with acto-myosin through its I/LWEQ domain.

### Preferential interaction of talin A with stretched actin filaments through its actin-binding domain

**Fig 6:**
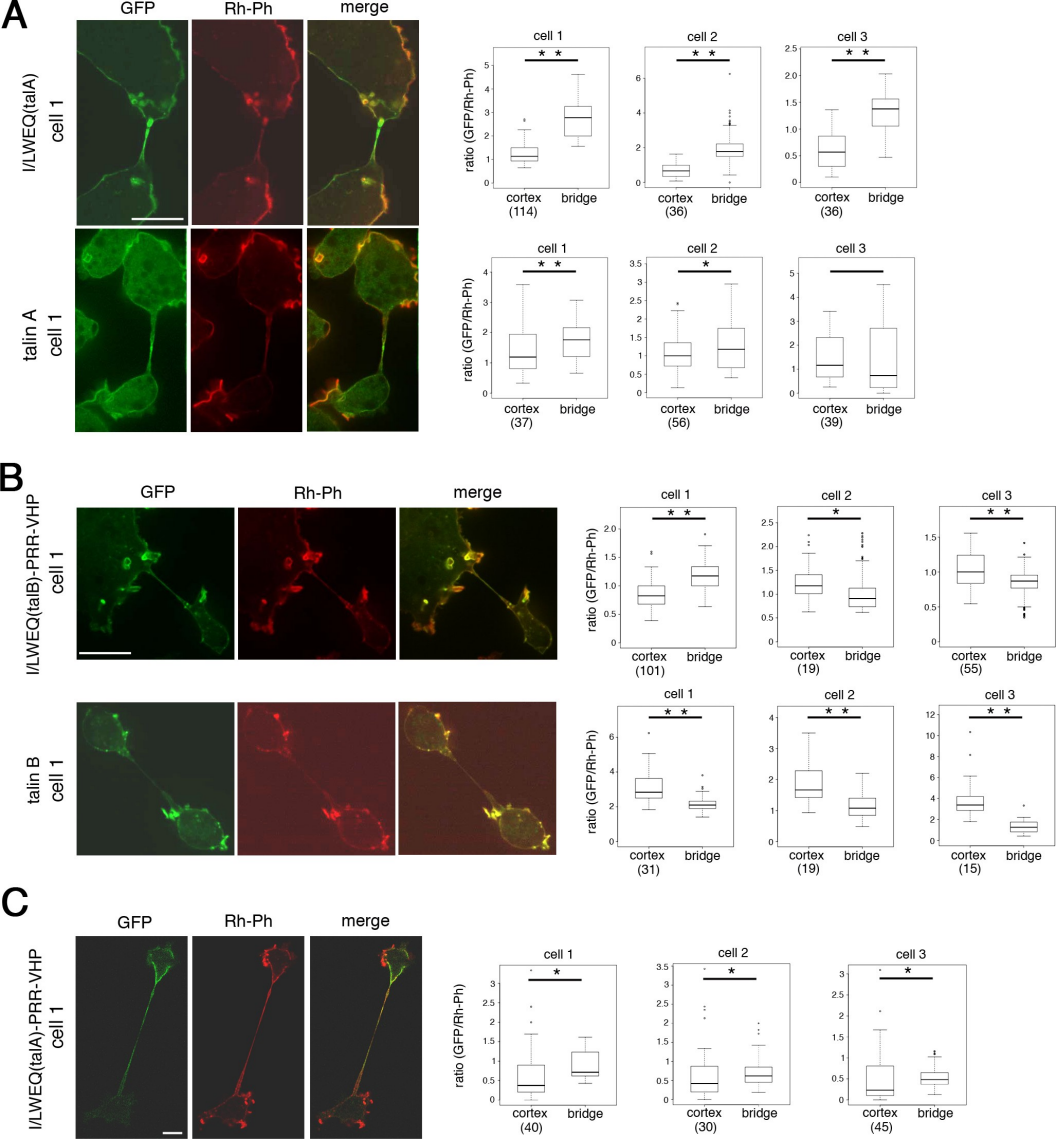
**Stretching actin filaments affect the localization of talin A, talin B, and their actin-binding domains.** Confocal images showing the distribution of GFP fusion proteins and actin filaments in dividing myosin II-/talin A-null cells expressing talin A-GFP, GFP-I/LWEQ(talA), and GFP-I/LWEQ(talA)-PRR-VHP (A,C, left), and dividing myosin II-/talin B-null cells expressing GFP-talin B or GFP-I/LWEQ(talB)-PRR-VHP (B, left). Cells of each line undergoing cytofission were fixed and stained with rhodamine-phalloidin (Rh-Ph) to visualize actin filaments. Merged images to demonstrate the relative abundance of GFP fusion proteins to actin filaments are also displayed. Only a small fraction of myosin II-/talin A-null cells expressing GFP-I/LWEQ(talA) underwent cytofission, consistent with our earlier report showing that efficient cytofission requires talin A [45]. Box plots show statistical analysis of three representative cells in each cell line, comparing fluorescence intensities of the GFP signals relative to the Rh-Ph signals between the cytoplasmic bridge and the cell cortex within one cell. Box plots for cell 1 of each line were obtained from the images shown on the left (cell 1). Images of cells 2 and 3 are shown in S3 Fig. Fluorescence intensities of GFP and Rh-Ph signals along the cytoplasmic bridge were measured by Image J software. For the cortical region, the fluorescence intensities in representative pixels along the plasma membrane were measured. Each representative pixel was within a distance of 10 pixels along the x or y axis to its nearest representative pixel and the total number of pixels measured for each cell, which depended on cell size, is indicated in parentheses in box plots. Boxes represent median +/- interquartile range and whiskers are confidence intervals that denote 10th-90th percentiles. Significance was tested using the two-sided Wilcoxon matched-pairs signed rank test. *: p < 0.05, **: p < 0.001. Scale bars: 10 μm.

The myosin II motor domain preferentially binds to stretched actin filaments *in vivo* [33]. We hypothesized that stretching of actin filaments by myosin II increased the affinity to I/LWEQ(talA), resulting in the co-localization of talin A with acto-myosin. We tested this hypothesis by examining whether talin A and I/LWEQ(talA) preferentially interacted with actin filaments stretched mechanically, even in the absence of myosin II. Myosin II-null cells become very large and harbor multiple nuclei in suspension culture due to the impairment of cytokinesis [41–43]. When attached to the substrate, however, the multinucleated cells immediately divide into smaller fragments by migration of cell portions in different directions [41–43]. This process was termed cytofission or cytokinesis C [43, 44]. Before the completion of fission, a thin cytoplasmic bridge connecting dividing fragments becomes highly elongated and stretched. We expressed talin A-GFP and GFP-I/LWEQ(talA) individually in cells lacking both myosin II and talin A (myosin II-/talin A-null cells), and determined whether these GFP fusion proteins preferentially localized along the cytoplasmic bridges where actin filaments are presumed to be stretched. The transformed cells placed on the substrate after suspension culture were fixed and permeabilized, followed by staining with rhodamine-phalloidin to visualize actin filaments. We quantified the relative fluorescence intensity of talin A-GFP or GFP-I/LWEQ(talA) by dividing the fluorescence intensity of GFP by that of rhodamine-phalloidin at each pixel, and compared the relative fluorescence intensity of GFP between the cytoplasmic bridge and the cell cortex within one cell. GFP-I/LWEQ(talA) clearly exhibited a stronger relative concentration along the cytoplasmic bridge than in the cortical region (Fig 6A and S3A Fig). Talin A-GFP also tended to be enriched along the cytoplasmic bridge, although the degree of enrichment was less than that of GFP-I/LWEQ(talA), and some dividing cells did not show a significant difference between the two regions (Fig 6A and S3B Fig). We therefore anticipate that co-localization of talin A with acto-myosin results from the preferential association of I/LWEQ(talA) with stretched actin filaments.

We also observed the localization of GFP-talin B and GFP-I/LWEQ(talB)-PRR-VHP in cells undergoing cytofission. As observed in talin B-null cells, the distribution of GFP-talin B was very similar to that of actin filaments in myosin II-/talin B-null cells expressing GFP-talin B. In other words, GFP-talin B strongly localized along rough peripheries and cup-shaped structures without pronounced accumulation along the cytoplasmic bridges during cytofission (Fig 6B and S3D Fig). GFP-I/LWEQ(talB)-PRR-VHP in myosin II-/talin B-null cells showed very similar localization patterns to GFP-talin B along the cortical region (Fig 6B and S3C Fig). Along the cytoplasmic bridges, however, its relative amount to actin filaments was larger than that of GFP-talin B and occasionally exceeded that of the cortical region (Fig 6B cell1). According to the results we have described thus far, the association with actin filaments through I/LWEQ(talB)-PRR-VHP appears to be a major determinant of talin B’s sub-cellular distribution, but it is not the only mechanism involved in the sub-cellular localization of talin B, as discussed in the next section.

We finally observed the localization of GFP-I/LWEQ(talA)-PRR-VHP in myosin II-/talin A-null cells undergoing cytofission. It exhibited higher enrichment along the cytoplasmic bridge than the cortex, which was qualitatively similar to GFP-I/LWEQ(talA) (Fig 6C and S3E Fig). However, the degree of enrichment of GFP-I/LWEQ(talA)-PRR-VHP was much weaker than that of GFP-I/LWEQ(talA) (Fig 6A and 6C and S3A and S3E Fig). Unlike the case of directed cell migration (Fig 4A), therefore, we found that the addition of the VHP domain blunted the localization specificity of I/LWEQ(talA).

### Exclusion of talin B from areas rich in stretched actin filaments

**Fig 7:**
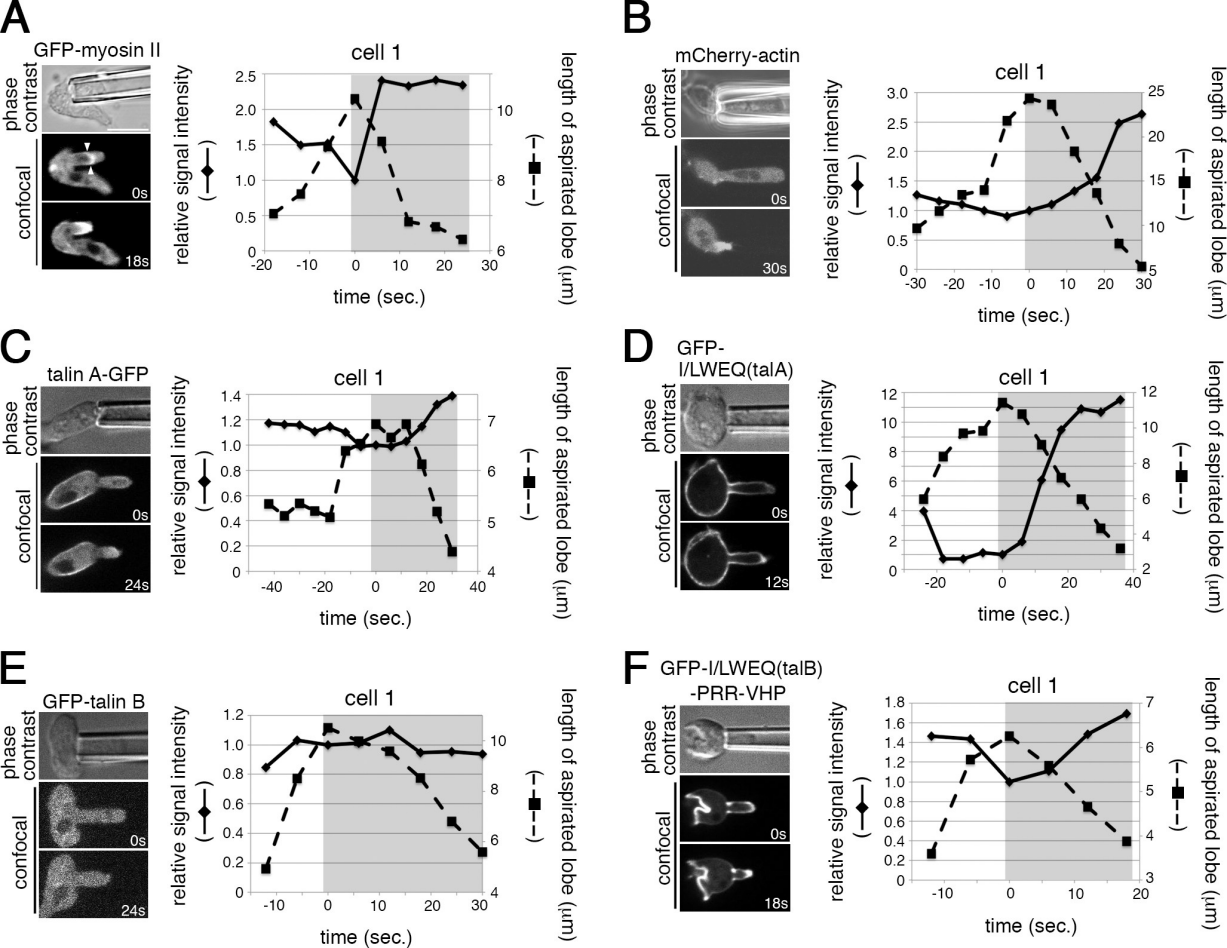
**Behaviors of talin A, talin B, and their actin-binding domains in aspiration assays.** (A-F, left) Phase contrast images showing cells aspirated by a micro-pipette (top) and the corresponding time lapse confocal images showing the responses of GFP-myosin II (A), mCherry-actin (B), talin A-GFP (C), GFP-I/LWEQ(talA) (D), GFP-talin B (E), and GFP-I/LWEQ(talB)-PRR-VHP (F) during sucking into the micro-pipette and the subsequent retraction of the aspirated projections. Times are indicated at lower right corners in seconds. The right panels show the time courses of relative fluorescence intensity changes of each GFP fusion protein at the tips of aspirated lobes (diamonds connected by solid lines) and changes in the lobe length (squares connected by broken lines) in a cell of each cell line. The time when the aspirated lobe was most elongated was taken as time zero, and the fluorescent intensities were normalized by their values at time zero. Shading shows the retraction phase. The data of two more cells in each cell line are shown in S4 Fig. Fluorescence intensities of GFP-myosin II, mCherry-actin, talin A-GFP, GFP-I/LWEQ(talA), and GFP-I/LWEQ(talB)-PRR-VHP, but not that of GFP-talin B, increased during the retraction of the lobe. The pair of arrowheads in (A) indicate the edges of the arc-shaped fluorescence signal representing the detached cortex. Scale bar: 5 μm.

We employed another experimental system to examine the effects of acto-myosin, in which actin filaments are presumed to be stretched, on the localization of talin A, talin B, and their actin-binding domains. When cells are sucked by a small micro-pipette, the aspirated lobe retracts by the contraction of acto-myosin redistributed in the deformed area, resulting in the accumulation of both actin and myosin II filaments in the retracting projection [14, 33, 46] (Fig 7A and 7B and S4A and S4B Fig). Talin A-GFP and GFP-I/LWEQ(talA), individually expressed in talin A-null cells, were moderately and markedly accumulated in the aspirated region, respectively, during the process of retraction (Fig 7C and 7D and S4C and S4D Fig). These behaviors are similar to that of myosin II. Similarly, GFP-I/LWEQ(talB)-PRR-VHP expressed in talin B-null cells accumulated in the retracting projection, probably reflecting the accumulation of actin filaments (Fig 7F and S4F Fig) [33]. In contrast, no GFP-talin B accumulation was detected in that region (Fig 7E and S4E Fig). These results further support the following hypothesis. Talin A strongly co-localizes with acto-myosin through the preferential binding between I/LWEQ(talA) and actin filaments stretched by myosin II. The non-selective binding of I/LWEQ(talB)-PRR-VHP with actin filaments is a major determinant of the sub-cellular localization of talin B. In addition, talin B appears to have an additional, unidentified mechanism by which it is excluded from regions rich in stretched actin filaments even though I/LWEQ(talB)-PRR-VHP is able to bind to them.

It is notable that signal intensities of the fluorescent proteins other than GFP-talin B at the tip regions decreased during elongation of the sucked projections. This was probably due to separation of the lipid bilayer from the underlying cell cortex there. The arc-shaped fluorescence signal, whose edges are indicated by a pair of arrowheads in Fig 7A, apparently represents the dissociated cortex after the plasma membrane was sucked into the pipette.

## Discussion

During directed cell migration, the extension and retraction of cell edges and assembly and disassembly of focal adhesions should be properly orchestrated. In *Dictyostelium*, talin A is enriched in the cell posterior to support the detachment from the substrate and the rear retraction [4]. In the present study, we demonstrated that talin B, the other talin homologue in *Dictyostelium*, localizes along the leading edge, which is in sharp contrast to talin A. Since talin is generally required for the formation of focal adhesions and may also link the cortical cytoskeleton to the plasma membrane [4, 13–15], talin B presumably fulfills those functions along the leading edge. Depletion of PKBR1, a protein kinase phosphorylating talin B, attenuates the attachment to substrates at the cell front [47], suggesting the possibility that talin B is one of the components involved in adhesion along the leading edge even though its depletion did not cause obvious phenotypic impairment in our experimental conditions. Furthermore, the binding ability of its FERM domain to PtdIns(3,4,5)P_3_, which concentrates within the plasma membrane of the leading edge [5], tempts us to speculate that talin B connects the cytoskeleton to the plasma membrane along the leading edge through simultaneous binding to actin filaments and PtdIns(3,4,5)P_3_.

In this study, we focused on the mechanism of the distinct sub-cellular distributions of talin A and talin B rather than their functions. We previously suggested two independent mechanisms that lead to the posterior enrichment of talin A during directed cell migration. In one mechanism, rearward cortical actin flow carries talin A to the rear. In the other mechanism, talin A is directly targeted to the posterior cortex by specific binding to acto-myosin [4]. In the present study, we revealed that talin A, but not talin B, is enriched along the cortical areas where myosin II is typically abundant, by comparing the sub-cellular localizations of talin A-RFP and GFP-talin B within the same non-polarized cells. Furthermore, in the uniform cAMP exposure experiments and the cell aspiration assays, both of which induced dynamic relocation of myosin II, talin A displayed relocation comparable to that of myosin II. These findings support a model arguing the direct targeting of talin A to acto-myosin. During fission of multinucleated myosin II-null cells, however, talin A tended to be predominantly associated with mechanically stretched actin filaments even in the absence of myosin II. We thus propose that talin A preferentially interacts with stretched actin filaments rather than with myosin II *per se*. I/LWEQ(talA) alone recapitulated the behaviors of talin A under all the experimental conditions described above, suggesting that I/LWEQ(talA) serves as a binding site to stretched actin filaments. Further experiments are needed to determine whether I/LWEQ(talA) binds to stretched actin filaments directly or indirectly mediated by some other actin-binding proteins. In aspiration assays and cytofission assays, the responses of I/LWEQ(talA) were augmented compared to those of full length talin A (Fig 6A and Fig 7C and 7D), suggesting negative regulation of the actin-binding domain in the whole structure of talin A. In the case of mouse talin1, its I/LWEQ domain is inhibited in the inactive form [48]. Furthermore, the large molecular size of talin A might impede its access to the long and thin space in the cytoplasmic bridge during cytofission. Altogether, we propose that the specific interaction of I/LWEQ(talA) with stretched actin filaments is an important mechanism responsible for the posterior recruitment of talin A during directed cell migration. This model is supported by the loss of posterior enrichment of talin A in polarized myosin II-null cells [4]. We previously demonstrated that the myosin II motor domain of *Dictyostelium* cells with a mutation enhancing the affinity to actin filaments was attracted to mechanically stretched actin filaments [33]. Behaviors of this myosin motor domain are similar to those of I/LWEQ(talA) in directed cell migration, myosin II independent cytofission, and cell aspiration assays [33].

Talin B was also suggested to require actin-containing structures for its sub-cellular localization, even though a biochemical assay demonstrated specific binding of the FERM domain of talin B to PtdIns(3,4,5)P_3_ (Fig 2). Talin B contains the I/LWEQ domain and the VHP domain in the C-terminal region, both of which are known as actin-binding domains [13, 26, 27]. Cosedimentation assays not only confirmed the actin-binding activity of this region (I/LWEQ(talB)-PRR-VHP) but also revealed its actin-bundling activity (Fig 3Ba). GFP-I/LWEQ(talB)-PRR-VHP was found to be localized along the leading edge in chemotaxing cells in a manner similar to the parent protein. In non-polarized cells, both talin B and I/LWEQ(talB)-PRR-VHP localized in rough sections of actin-rich cortex. In starved cells uniformly stimulated with cAMP, their temporary relocation to the cortical area coincided with the transient occurrence of actin polymerization there. Based on these observations, the amount of actin filaments appears to be a major factor determining the sub-cellular distribution of talin B, and I/LWEQ(talB)-PRR-VHP is likely to mediate this association. Additionally, other actin-binding proteins may also be involved in the interaction of I/LWEQ(talB)-PRR-VHP with actin filaments. For example, the proline-rich region residing between I/LWEQ(talB) and VHP typically confers binding sites to the SH3 domain, which is contained in multiple proteins as a docking site [49].

In general, low affinity to actin filaments may enhance anterior localization of actin-binding proteins during directed cell migration. This is because this low affinity allows those proteins to dissociate rapidly from actin filaments before they are transported to the rear by rearward cortical actin flow and thereby maintains a high concentration of cytoplasmic pool available for binding to newly formed actin filaments in the anterior region. This notion is supported by the fact that certain actin-binding domains with an increased affinity, either by dimerization or multimerization, show a more posterior localization than those in a monomeric state [40]. However, this does not appear to be the case with talin B according to individual characterization of I/LWEQ(talB) and VHP. Although GFP-I/LWEQ(talB) localized along the lagging edge, addition of the VHP domain to the C-terminus of I/LWEQ(talB) altered its localization to the leading edge even though the addition of VHP dramatically increased the affinity to actin filaments in cosedimentation assays. Since GFP-PRR-VHP localized along the leading edge, it may be that the VHP domain somehow recognizes a specific feature of newly formed actin filaments with high affinity, so that VHP determines the sub-cellular localization of I/LWEQ(talB)-PRR-VHP in the actin-rich anterior region. Nonetheless, the addition of VHP maintained the posterior localization of I/LWEQ(talA) (Fig 4A), although it affected the distribution in cytofission (Figs 6A, 6C and S3A and S3E). Further investigation will be required to thoroughly elucidate the difference between I/LWEQ(talA) and I/LWEQ(talB).

Unlike talin A and its I/LWEQ domain (I/LWEQ(talA)), talin B and I/LWEQ(talB)-PRR-VHP showed different distribution patterns under certain situations. In dividing myosin II-null cells, GFP-I/LWEQ(talB)-PRR-VHP was present along the cytoplasmic bridges, but GFP-talin B was not. The cell aspiration assays revealed more clearly different behaviors between talin B and I/LWEQ(talB)-PRR-VHP. The retracting projection showed the accumulation of GFP-I/LWEQ(talB)-PRR-VHP as in talin A-GFP and GFP-I/LWEQ(talA). In contrast, GFP-talin B itself never concentrated there. Although we hypothesize that actin-binding of I/LWEQ(talB)-PRR-VHP would be one of the factors to direct the localization of talin B, there is an additional, unidentified mechanism that excludes talin B from regions where stretched actin filaments are abundant, enhancing the anterior restriction of talin B during directed cell migration. The FERM domain of talin generally provides binding sites not only for membrane lipids but also for multiple talin-binding proteins, and the central rod region also binds several proteins such as vinculin and actin filaments [13]. These functional domains may be additionally involved in the localization of full-length talin B as well.

Since actin filaments harbor multiple conformations [50, 51], their structures are most likely different between the leading and lagging edges. Especially, tensile forces reportedly change the conformations of actin subunits [52–54], and this could confer the specific recognition sites for the I/LWEQ domain of talin A, as well as the mutant form of the myosin II motor domain [33]. In contrast, cofilin, an actin depolymerizing protein, preferentially associates with relaxed actin filaments in mammalian cells, providing another example of the recognition of a specific structure of actin filaments by an actin-binding protein [55]. Since *Dictyostelium* cofilin accumulates in the anterior region, actin filaments there could possess a structural feature that attracts certain actin-binding proteins, including cofilin and talin B [56, 57]. Alternatively, differences between higher order structures composed of actin filaments may direct the specific localization of talin A and talin B. Along the leading edge, individual actin filaments are more or less oriented towards the plasma membrane pushing the leading edge forward by the elongation of the growing ends. In the remainder of the cortical region, actin filaments lie parallel to the plasma membrane. In those actin structures, various proteins accumulating either in the leading or lagging areas affect geometries of actin filaments in various manners, such as cross-linking to form an orthogonal meshwork and bundling to form parallel or antiparallel bundles. These elements might present architectural differences of actin structures recognized by the dimeric actin-binding regions including talin A and talin B. The results of cosedimentation assays and sub-cellular localization analysis demonstrated coupling between the anterior localization and the actin-bundling activity in I/LWEQ(talB)-PRR-VHP. Also in mouse talin1, a specific segment of the I/LWEQ module is responsible for both the targeting to focal adhesions and actin-bundling activity [16], resulting in the coupling between the two. Modulation of actin structures might be an important factor to determine the sub-cellular localizations of actin-binding proteins.

Mammalian talin1 and talin2 tend to accumulate in peripheral and central focal adhesions, respectively, although the mechanism underlying their distinct sub-cellular localization has not been clarified [21]. Mechanical tension imposed by stress fibers, contractile bundles composed of actin and myosin II filaments, is higher in peripheral than in central focal adhesions [58]. This is consistent with the enrichment of myosin II around peripheral regions [58, 59]. Intriguingly, the I/LWEQ domain of murine talin1 is required for promoting the higher tension in peripheral focal adhesions than in central focal adhesions [60], implying that it binds to stress fibers under high tension. These findings tempt us to speculate that C-termini of the two mammalian talins also have different affinities for stretched actin filaments, resulting in their distinct sub-cellular localizations.

## Conclusion

We propose that specific associations of actin-binding domains with distinct actin-containing structures are one of the important mechanisms to direct specific localizations of actin-binding proteins, including talin A and talin B. This proposed mechanism is specifically important for maintaining their distinct distributions during directed cell migration.

## Supporting information

S1 Fig Expression of GFP-talin B in talin B-null cells. (A) Immuno-blot analysis was applied to total extracts of wild-type, talin B-null, and talin B-null cells transformed with the GFP-talin B construct. (Left) The anti-talin B antiserum detected a band with a predicted size of talin B in the extract of wild-type cells and a band with a slightly larger size in the extract of the talin B-null transformant, whereas no bands were detected in the extract of talin B-null cells. (Middle) The anti-GFP antibody detected a band with a predicted size of GFP-talin B only in the lysate of the talin B-null transformant. (Right) Expression levels of actin were used as a loading control. Marker sizes (kDa) are indicated on the left sides of the blots. Bands of talin B and GFP-talin B are indicated by asterisks. These results confirmed the expression of GFP-talin B in the talin B-null transformant. (B) A phase contrast image of talin B-null cells transformed with the GFP-talin B construct (left) and the fluorescence image of the same field (right). In the phase contrast image, cells showing the fluorescence signal are indicated by arrows. We determined the fraction of fluorescent cells by counting them, and found that 62% of cells exhibited the fluorescence signal (107 out of 170 cells). Scale bar: 10 μm.
(TIF)

S2 Fig Alignment of the I/LWEQ domains and sub-cellular localizations of talins’ C-terminal fragments. (A) Alignment of the I/LWEQ domains of talin A and talin B was performed by the clustalW program. Asterisks indicate identical amino acids. Colons and periods indicate strongly and weakly similar amino acids, respectively. Conserved amino acids supposed to be important for dimerization in vertebrate talins are shown in red. Numbers represent the initial and last amino acid positions of each I/LWEQ domain. (B) Confocal images of streaming wild-type cells expressing GFP-PRR-VHP (left) or GFP-I/LWEQ(talA)-PRR-VHP (right). Arrows indicate the direction of migration. Scale bar: 10 μm.
(TIF)

S3 Fig Confocal images of cytokinesis C. Confocal images showing the distribution of GFP fusion proteins and actin filaments in dividing myosin II-/talin A-null cells expressing GFP-I/LWEQ(talA), talin A-GFP, or GFP-I/LWEQ(talA)-PRR-VHP (A,B,E), and dividing myosin II-/talin B-null cells expressing GFP-I/LWEQ(talB)-PRR-VHP or GFP-talin B (C,D). Those ten cells were subjected to statistical analyses shown in Fig 6. Scale bars: 10 μm.
(TIF)

S4 Fig Quantification of aspiration assays. Time courses of fluorescence intensity changes (diamonds) of GFP-myosin II (A), mCherry-actin (B), talin A-GFP (C), GFP-I/LWEQ(talA) (D), GFP-talin B (E), and GFP-I/LWEQ(talB)-PRR-VHP (F) at the tips of retracting lobes and changes in the lobe length (squares) were determined for each experiment. Shaded areas indicate the period of the lobe retraction. These data accompany Fig 7. Scale bar: 5 μm.
(TIF)

S1 File Raw data to build graphs in Fig 6. (XLSX)

S2 File Raw data to build graphs in Fig 7. (XLSX)

S3 File Raw data to build graphs in S4 Fig. (XLSX)

S1 Movie Time-lapse images of streaming talin B-null cells expressing GFP-talin B. Fluorescent images were captured by confocal microscopy at 5-s intervals
(AVI)

S2 Movie Time-lapse images of streaming talin B-null cells expressing GFP-I/LWEQ(talB)-PRR-VHP. Fluorescent images were captured by confocal microscopy at 5-s intervals.
(AVI)

S3 Movie Time-lapse images of streaming talin B-null cells expressing GFP-I/LWEQ(talB). Fluorescent images were captured by confocal microscopy at 5-s intervals.
(AVI)

S4 Movie Time-lapse images of streaming talin B-null cells expressing GFP-PRR-VHP. Fluorescent images were captured by confocal microscopy at 5-s intervals.
(AVI)

S5 Movie Time-lapse images of streaming talin A-null cells expressing GFP-I/LWEQ(talA)-PRR-VHP. Fluorescent images were captured by confocal microscopy at 5-s intervals.
(AVI)
