# Supplementary figures and images for: Actin-binding domains mediate the distinct distribution of two Dictyostelium Talins through different affinities to specific subsets of actin filaments during directed cell migration
Source: PLoS One. 2019 Apr 4;14(4):e0214736. doi: 10.1371/journal.pone.0214736 (PMC6449030; doi:10.1371/journal.pone.0214736)

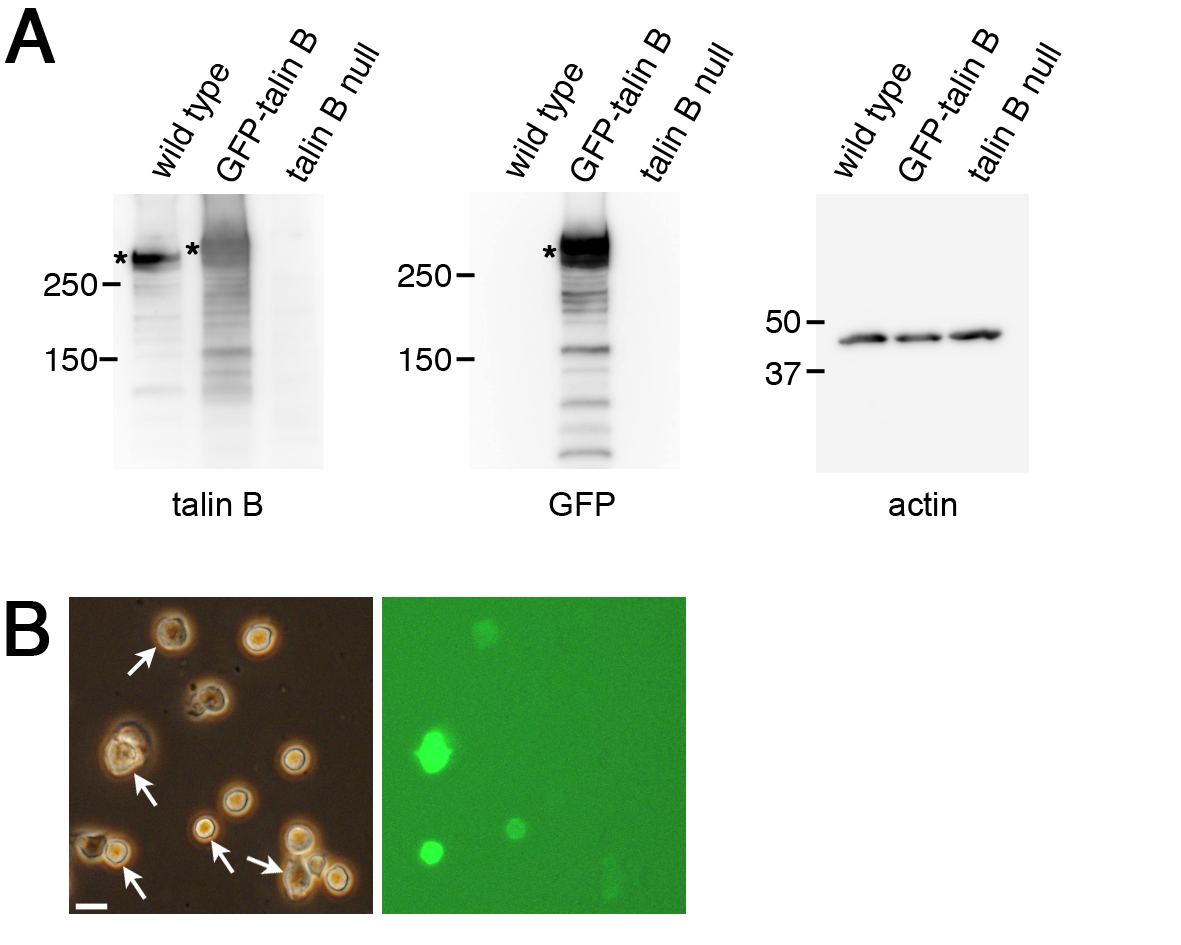

Supplement: S1 Fig — (A) Immuno-blot analysis was applied to total extracts of wild-type, talin B-null, and talin B-null cells transformed with the GFP-talin B construct. (Left) The anti-talin B antiserum detected a band with a predicted size of talin B in the extract of wild-type cells and a band with a slightly larger size in the extract of the talin B-null transformant, whereas no bands were detected in the extract of talin B-null cells. (Middle) The anti-GFP antibody detected a band with a predicted size of GFP-talin B only in the lysate of the talin B-null transformant. (Right) Expression levels of actin were used as a loading control. Marker sizes (kDa) are indicated on the left sides of the blots. Bands of talin B and GFP-talin B are indicated by asterisks. These results confirmed the expression of GFP-talin B in the talin B-null transformant. (B) A phase contrast image of talin B-null cells transformed with the GFP-talin B construct (left) and the fluorescence image of the same field (right). In the phase contrast image, cells showing the fluorescence signal are indicated by arrows. We determined the fraction of fluorescent cells by counting them, and found that 62% of cells exhibited the fluorescence signal (107 out of 170 cells). Scale bar: 10 μm. (TIF) [file pone.0214736.s001.tif]

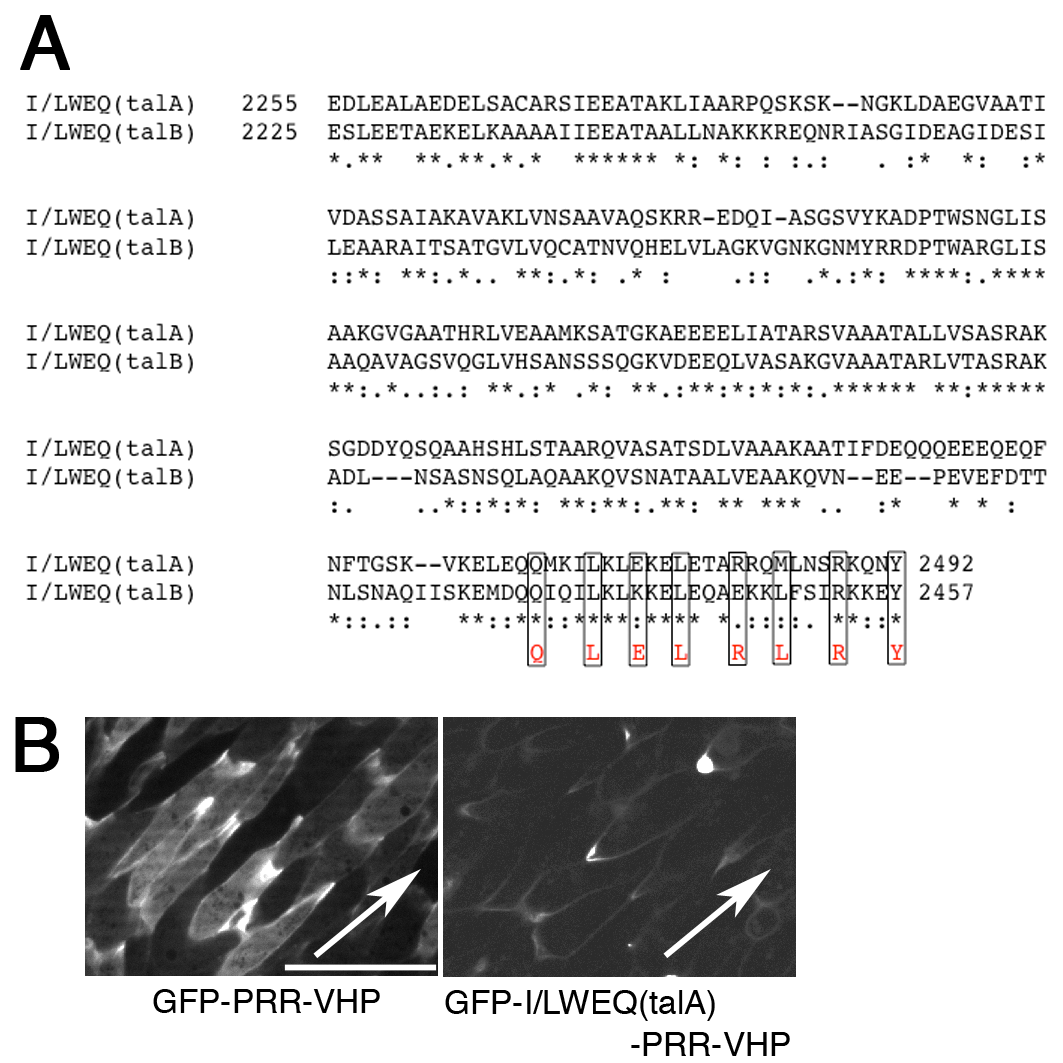

Supplement: S2 Fig — (A) Alignment of the I/LWEQ domains of talin A and talin B was performed by the clustalW program. Asterisks indicate identical amino acids. Colons and periods indicate strongly and weakly similar amino acids, respectively. Conserved amino acids supposed to be important for dimerization in vertebrate talins are shown in red. Numbers represent the initial and last amino acid positions of each I/LWEQ domain. (B) Confocal images of streaming wild-type cells expressing GFP-PRR-VHP (left) or GFP-I/LWEQ(talA)-PRR-VHP (right). Arrows indicate the direction of migration. Scale bar: 10 μm. (TIF) [file pone.0214736.s002.tif]

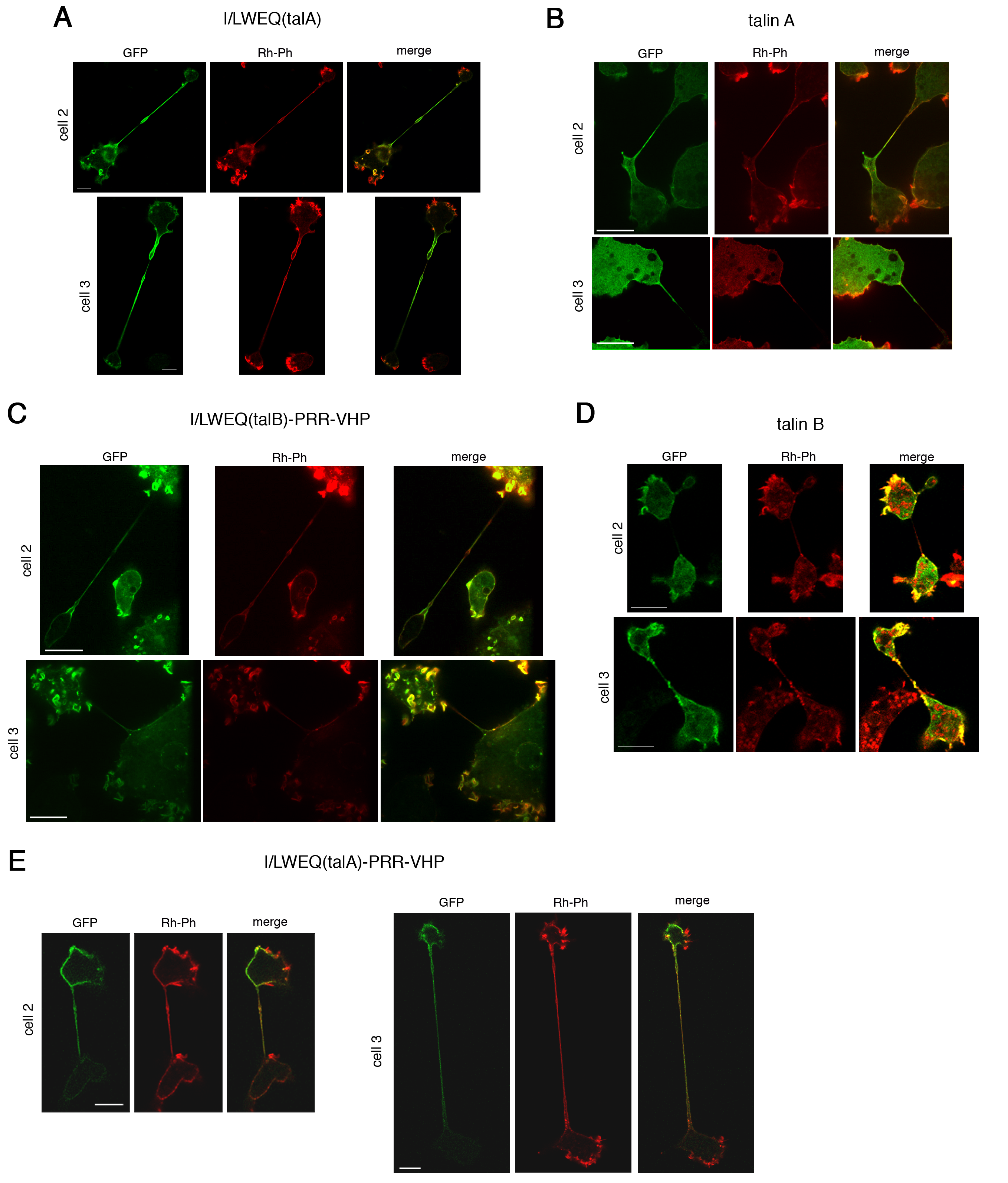

Supplement: S3 Fig — Confocal images showing the distribution of GFP fusion proteins and actin filaments in dividing myosin II-/talin A-null cells expressing GFP-I/LWEQ(talA), talin A-GFP, or GFP-I/LWEQ(talA)-PRR-VHP (A,B,E), and dividing myosin II-/talin B-null cells expressing GFP-I/LWEQ(talB)-PRR-VHP or GFP-talin B (C,D). Those ten cells were subjected to statistical analyses shown in Fig 6. Scale bars: 10 μm. (TIF) [file pone.0214736.s003.tif]

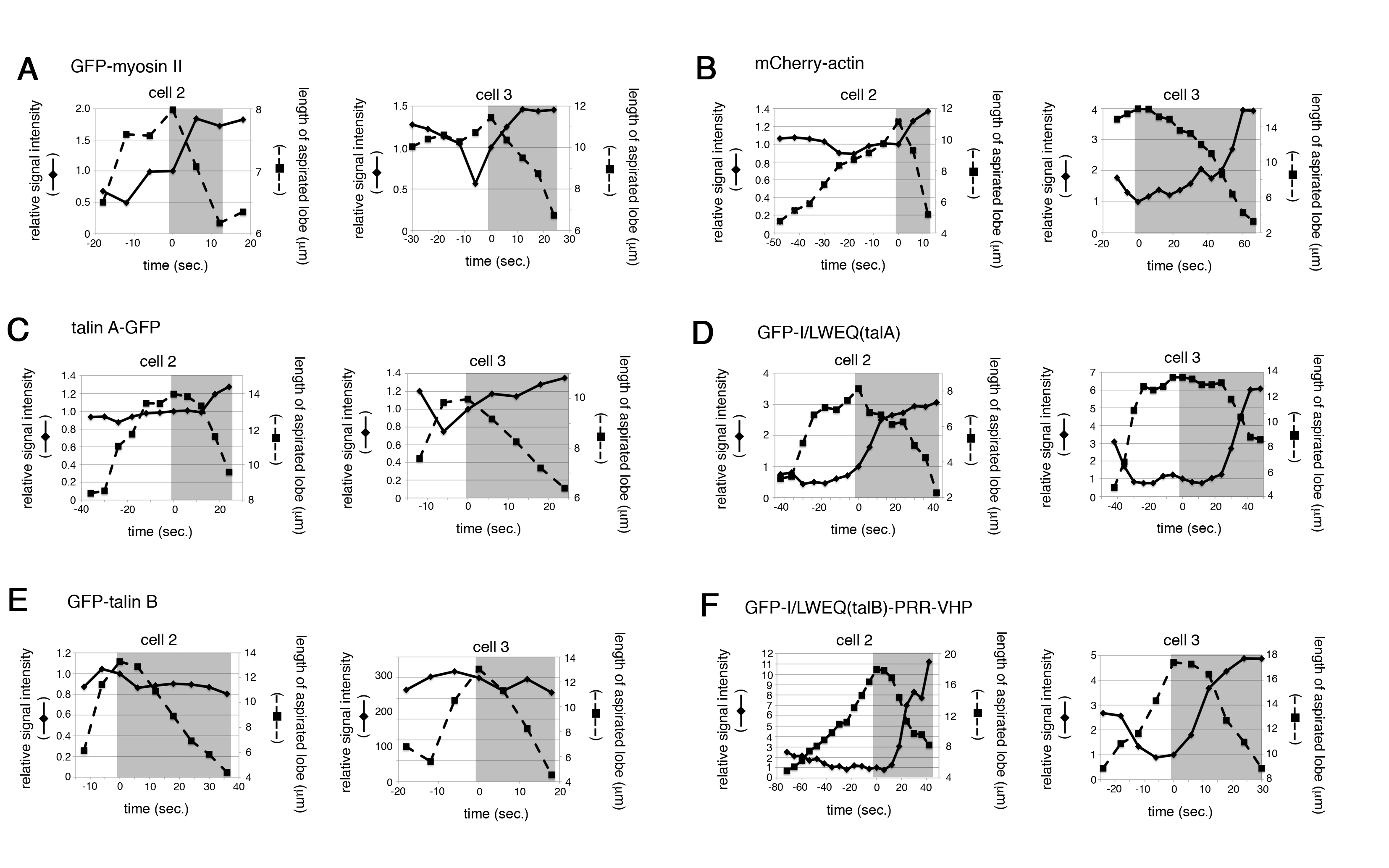

Supplement: S4 Fig — Time courses of fluorescence intensity changes (diamonds) of GFP-myosin II (A), mCherry-actin (B), talin A-GFP (C), GFP-I/LWEQ(talA) (D), GFP-talin B (E), and GFP-I/LWEQ(talB)-PRR-VHP (F) at the tips of retracting lobes and changes in the lobe length (squares) were determined for each experiment. Shaded areas indicate the period of the lobe retraction. These data accompany Fig 7. Scale bar: 5 μm. (TIF) [file pone.0214736.s004.tif]
